# Dechlorination of three tetrachlorobenzene isomers by contaminated harbor sludge-derived enrichment cultures follows thermodynamically favorable reactions

**DOI:** 10.1007/s00253-016-8004-8

**Published:** 2016-12-01

**Authors:** Yue Lu, Javier Ramiro-Garcia, Pieter Vandermeeren, Steffi Herrmann, Danuta Cichocka, Dirk Springael, Siavash Atashgahi, Hauke Smidt

**Affiliations:** 1 Laboratory of Microbiology, Wageningen University, Wageningen, The Netherlands; 2 College of Environmental Science and Engineering, Hunan University, Changsha, People’s Republic of China; 3 Laboratory of Systems and Synthetic Biology, Wageningen University, Wageningen, The Netherlands; 4 Division of Soil and Water Management, KU Leuven, Leuven, Belgium

**Keywords:** Tetrachlorobenzenes, Organohalide respiration, *Dehalococcoides mccartyi*, *Dehalobacter*, Thermodynamically favorable reactions

## Abstract

Dechlorination patterns of three tetrachlorobenzene isomers, 1,2,3,4-, 1,2,3,5-, and 1,2,4,5-TeCB, were studied in anoxic microcosms derived from contaminated harbor sludge. The removal of doubly, singly, and un-flanked chlorine atoms was noted in 1,2,3,4- and 1,2,3,5-TeCB fed microcosms, whereas only singly flanked chlorine was removed in 1,2,4,5-TeCB microcosms. The thermodynamically more favorable reactions were selectively followed by the enriched cultures with di- and/or mono-chlorobenzene as the main end products of the reductive dechlorination of all three isomers. Based on quantitative PCR analysis targeting 16S rRNA genes of known organohalide-respiring bacteria, the growth of *Dehalococcoides* was found to be associated with the reductive dechlorination of all three isomers, while growth of *Dehalobacter*, another known TeCB dechlorinator, was only observed in one 1,2,3,5-TeCB enriched microcosm among biological triplicates. Numbers of *Desulfitobacterium* and *Geobacter* as facultative dechlorinators were rather stable suggesting that they were not (directly) involved in the observed TeCB dechlorination. Bacterial community profiling suggested bacteria belonging to the phylum *Bacteroidetes* and the order *Clostridiales* as well as sulfate-reducing members of the class *Deltaproteobacteria* as putative stimulating guilds that provide electron donor and/or organic cofactors to fastidious dechlorinators. Our results provide a better understanding of thermodynamically preferred TeCB dechlorinating pathways in harbor environments and microbial guilds enriched and active in anoxic TeCB dechlorinating microcosms.

**Electronic supplementary material:**

The online version of this article (doi:10.1007/s00253-016-8004-8) contains supplementary material, which is available to authorized users.

## Introduction

Chlorobenzenes (CBs) are aromatic chlorinated compounds with a benzene ring substituted with one to six chlorine atoms. CBs are important intermediates in industrial synthesis of pesticides and bulk chemicals (Barber et al. 2005; Malcolm et al. 2004). As a result of massive use and uncontrolled disposal on one hand and high persistence and bioaccumulation in ecosystems on the other hand, broad distribution of these pollutants has been found in a range of different environments (Barber et al. 2005; Malcolm et al. 2004; Wang et al. 2010). Partial reductive dechlorination of highly chlorinated benzenes has been shown in a broad range of sub-oxic/anoxic environments such as soil (Jiang et al. 2015; Ramanand et al. 1993), riverbed sediment (Nowak et al. 1996; Taş et al. 2010; Taş et al. 2011), sewage sludge (Fathepure et al. 1988; Fennell et al. 2004), and drainage ditch (Nelson et al. 2014) with lower chlorinated benzenes as end products. Even though monochlorobenzene (MCB) was considered to be recalcitrant to biotransformation in anoxic environments (Field and Sierra-Alvarez 2008), several studies reported further dechlorination of MCB to benzene in anaerobic enrichment cultures originating from freshwater sediment samples (Fung et al. 2009; Nowak et al. 1996; Zhou et al. 2015). TeCBs are important intermediates of HCB dechlorination, and include three isomers, i.e., 1,2,3,4-, 1,2,3,5-, and 1,2,4,5-TeCB. TeCBs can be reductively dechlorinated following diverse pathways (Adrian and Görisch 2002; Field and Sierra-Alvarez 2008). Preference for thermodynamically more favorable CB reductive dechlorination pathways was shown before by a microbial consortium originating from lake sediment (Beurskens et al. 1994), but this selectivity was not observed in other microcosms derived from contaminated freshwater sediment samples (Vandermeeren et al. 2014; Zhou et al. 2015).

Organohalide respiration (OHR), i.e., energy conservation from respiratory reductive dechlorination of organohalides as the final electron acceptors, is currently the only known metabolic microbial process for the partial/complete dechlorination of highly chlorinated benzenes like hexachlorobenzene (HCB), pentachlorobenzene (QCB), and tetrachlorobenzenes (TeCBs) under anoxic conditions (Adrian and Görisch 2002; Field and Sierra-Alvarez 2008). Therefore, the presence and activity of organohalide respiring bacteria (OHRB) is pivotal for bioremediation of anoxic CB-contaminated environments. Currently known bacterial isolates able to dechlorinate TeCB isomers belong to *Dehalococcoides mccartyi* in the phylum *Chloroflexi* (Adrian et al. 2000; Fennell et al. 2004) and *Dehalobacter* in the phylum *Firmicutes* (Nelson et al. 2014). *D. mccartyi* strains CBDB1 (Adrian et al. 2000), 195 (Fennell et al. 2004) and DCMB5 (Pöritz et al. 2015) dehalogenate CB congeners with four or more chlorine substitutes to trichlorobenzenes (TCBs) or dichlorobenzenes (DCBs). Besides, *Dehalobacter* spp. strains 12DCB1 and 13DCB1 and a highly enriched culture containing *Dehalobacter* sp. 14DCB1 were shown to dechlorinate highly chlorinated CBs to DCBs and MCB (Nelson et al. 2014). In addition to these obligate organohalide respiring isolates that as to the best of our knowledge, are restricted to OHR as the sole metabolism to conserve energy, evidence for the growth of members of metabolically versatile OHRB in the presence of CBs was reported. Bacteria of the genus *Geobacter* were found associated with HCB dechlorination in an anaerobic consortium also containing *Dehalococcoides* and originating from contaminated river sediment as determined by genus-specific quantitative PCR (qPCR), suggesting members of the genus *Geobacter* also may serve as CB dechlorinators (Zhou et al. 2015). Furthermore, a recently reported bacterial community analysis with 454 pyrosequencing of 16S ribosomal RNA (rRNA) gene fragments conducted on a range of CB-dechlorinating enrichments from contaminated river sludge revealed a high relative abundance (21–55%) of populations closely related to *Desulfitobacterium*, while 16S rRNA genes of *Dehalococcoides* and *Dehalobacter* together represented less than 0.53% of the bacterial community (Vandermeeren et al. 2014).

Up to this date, the fate of TeCBs in marine and estuarine environments is still not well known. 1,2,4-TCB dechlorination in sediment collected from Ise Bay (Japan) revealed that DCB production rates were related to anoxic plate count numbers and activity of sulfate reducing bacteria, although there was no solid evidence showing those sulfate reducers have direct involvement in 1,2,4-TCB dechlorination (Yonezawa et al. 1994). Furthermore, dechlorination of CBs was reported in enrichments derived from estuarine sediments collected in Tsurumi river (Japan) (Masunaga et al. 1996) and Bayou d’Inde (USA) (Pavlostathis and Prytula 2000), but the microorganisms responsible for dechlorination were not identified. To date, the only reported CB-dechlorinating bacterium isolated from an estuarine environment (Charleston Harbor, USA) is strain DF-1, later re-classified as *Dehalobium chlorocoercia* (Löffler et al. 2013), which was shown to dechlorinate HCB via 1,2,3,5-TeCB to 1,3,5-TCB (Wu et al. 2002). This pathway was also proposed as the main pathway of dechlorination of HCB and QCB in estuarine sediments with very low levels of other CBs detected (Pavlostathis and Prytula 2000; Wu et al. 2002). Overall, dechlorination of CBs and in particular of TeCB in marine and estuarine sediments remains poorly explored, and better understanding of the activity of CB-dehalogenating microbes and the microbial context they interact with in those habitats is required for their in situ bioremediation (Zanaroli et al. 2015).

The aim of this study was to assess the following: (i) the TeCB dechlorination potential of microbial communities present in an estuarine environment, (ii) microbial community dynamics during anoxic cultivation, and (iii) the preferred TeCB dechlorinating pathways. To this end, we set up TeCB-fed anoxic microcosms with sludge derived from a harbor contaminated with tributyltin, mineral oil, and polyaromatic hydrocarbons. A combination of bacterial community profiling and qPCR was applied to gain better insight into TeCB dechlorinators and co-existing, non-dechlorinating bacterial guilds that may be important for sustained dechlorination and therefore aid in improving in situ bioremediation.

## Materials and methods

### Chemicals

CBs, benzene, and 1,3,5-tribromobenzene (TBrB) of analytical grade and high purity were purchased from Sigma Aldrich. Benzene, MCB, 1,2-DCB, 1,4-DCB, 1,2,3-TCB, 1,2,4-TCB, 1,3,5-TCB, and 1,2,3,5-TeCB had a purity of 99%; 1,3-DCB, 1,2,4,5-TeCB and 1,3,5-TBrB 98%; and 1,2,3,4-TeCB 96%.

### Microcosm setup

**Fig. 1:**
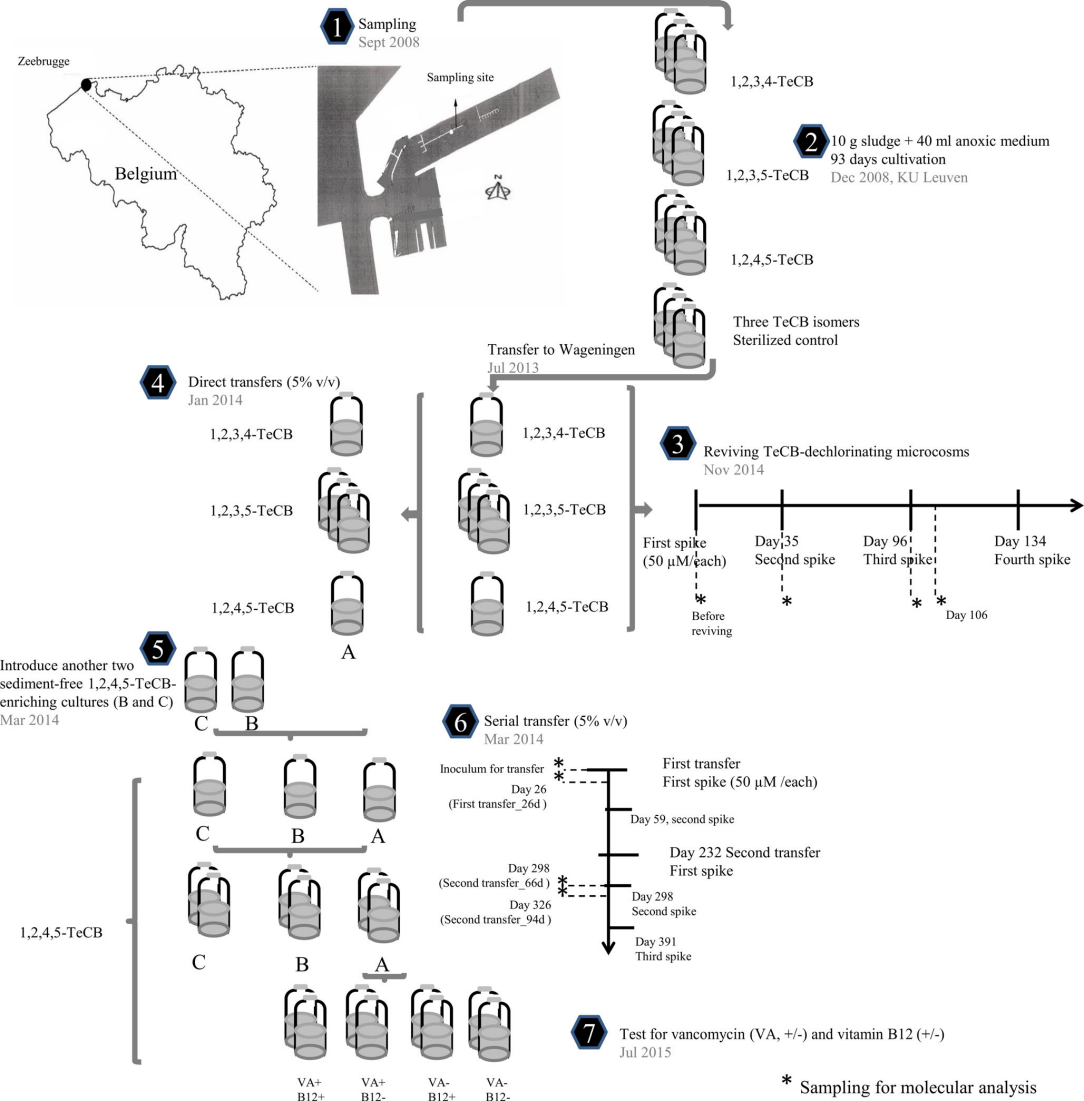
Schematic representation of the experimental strategy

Harbor sludge from Zeebrugge, Belgium was sampled (Fig. 1, step 1) to prepare anoxic microcosms. All samples were collected in September 2008, and stored for 3 months at 4 °C until they were used in this study. The microcosms were set up in triplicates as described elsewhere (Vandermeeren et al. 2014) with 10 g of sludge and 40 ml of an anoxic and sterilized medium with 2 g/L yeast extract (Holliger et al. 1998). One spike of lactate (20 mM) was added as electron donor and two spikes of TeCB isomers were added from acetone stocks to final concentrations of 50 μM for each isomer where necessary and microcosms were incubated non-shaking at 25 °C in the dark for a period of 93 days (Fig. 1, step 2). After dechlorination of the first TeCB spike to TCBs and DCBs, a second spike (50 μM) of the corresponding TeCB isomer was supplied to the cultures. As residual lactate concentrations were considered sufficient, lactate was not replenished with the second TeCB spike. Abiotic controls were prepared by addition of 0.5 ml of 37% formaldehyde. Cultures were subsequently kept for 5 years at 4 °C, after which a subset (single 1,2,3,4- and 1,2,4,5-TeCB enrichment cultures and triplicate 1,2,3,5-TeCB cultures) was transferred to Wageningen, the Netherlands, for further characterization of TeCB dechlorination. To revive TeCB dechlorination in the original cultures, 20 mM lactate and 30 ml of fresh anoxic medium was added to the original bottles. Bottles were sealed with a viton stopper, the headspace was exchanged with N_2_/CO_2_ (80:20 *v*/v), and the respective TeCBs (50 μM/each in acetone) were added. The dechlorinating cultures were amended with the respective TeCBs and lactate as mentioned on day 35, 96, and 134 of incubation. All cultures were maintained actively dechlorinating for over 200 days without shaking at 25 °C in the dark (Fig. 1, step 3). To ensure efficient electron donor and micronutrients supply, 20 mM lactate and 42 ml fresh anoxic medium were added to the cultures on day 245 of incubation.

To study the dynamics of degradation pathways and microbial communities and to obtain sediment-free TeCB-enriched cultures, serial transfers were performed. A 5% inoculum from the original TeCB-fed microcosms was transferred into anoxic bottles containing growth medium, fed with 50 μM of the same TeCB isomer as the initial inoculum and cultivated for 66 days (Fig. 1, step 4). Further transfers were conducted with the 1,2,4,5-TeCB dechlorinating culture enriched from harbor sludge (culture A in Fig. 1, step 5), and their performance was compared to that of transfers of two sediment-free 1,2,4,5-TeCB-enriching cultures (cultures B and C in Fig. 1, step 5) that were previously obtained from chlorinated aromatics-contaminated river sludge collected in Kanaal Ieper-Ijzer, Belgium (Vandermeeren et al. 2014). A 5% inoculum was taken for serial transfers (Fig. 1, step 6).

Effects of vancomycin addition and/or vitamin B12 starvation on the dechlorination activity of the sediment-free 1,2,4,5-TeCB-fed enrichments were investigated with a 2 × 2 factorial design experiment (Fig. 1, step 7) that was set up in duplicate. Twelve microgram per liter of vancomycin and 0.03 μM of vitamin B12 were added as required.

### Detection of benzene and chlorinated benzenes

Benzene and CBs were quantified on a GC-2000 equipped with flame ion detector (FID) (Shimadzu, Japan) and an Rxi-5Sil capillary column (RESTEK, USA). 0.2 ml headspace samples were used for benzene measurement as described before (Liang et al. 2011), and 1 ml liquid samples were used for the detection of CBs. Liquid samples were extracted with 400 μl hexane by overnight shaking at 600 rpm followed by 100 μl hexane phase transfer to HPLC vials containing 5 μl of 1 mM 1,3,5-tribromobenzene dissolved in hexane as an internal standard. The column temperature program was 60 °C held for 4 min, followed by a gradient of 5 °C min^−1^ to 180 °C and another held for 3 min. The carrier gas was nitrogen at a flow of 1 ml min^−1^, a split flow of 12 ml min^−1^ with a split ratio of 1:10. Injection was performed by an auto-sampler (Shimadzu, Japan) injecting 2 μl of the sample into a split injector held at 250 °C. Standards with a mixture of benzene and CBs at 10, 20, 40, 60, 100, and 200 μM were set up in 125 ml serum bottles with the same headspace-liquid phase ratio of treatment cultures.

### Sampling for molecular analysis and DNA extraction

Cultures were sampled for molecular analysis as shown in Fig. 1. The samples taken before adding TeCBs to the original bottles were indicated as ‘Before reviving’ (Fig. 1, step 3). Two milliliters of slurry or liquid samples were withdrawn from each bottle, centrifuged (5417R, Eppendorf, Germany) at 6010 *g* for 5 min at 4 °C, and pellets were stored at −20 °C. DNA isolation was performed using the PowerSoil DNA isolation kit (MO-BIO, USA) according to the manufacturer’s instructions except that three 45 s bead beating (5.5 m/s) steps using a Fastprep Instrument (MP Biomedicals, USA) were included instead of the horizontally vortexing step suggested in the manual. The quality and quantity of DNA was checked using a Nanodrop 2000c Spectrophotometer (Thermo Fisher Scientific, USA) and agarose gel electrophoresis.

### Illumina MiSeq sequencing and data analysis

A 2-step PCR strategy was used to generate barcoded amplicons from the V1-V2 region of the 16S rRNA gene. The first PCR (50 μl) contained 1× HF buffer (Thermo Scientific™, The Netherlands), 1 μl dNTP Mix (10 mM; Promega, The Netherlands), 1 U of Phusion® Hot Start II High-Fidelity DNA polymerase (Thermo Scientific™), 500 nM of 27F–DegS forward primer, 500 nM of 338R I and II reverse primers (Table S1), and 1 μL template DNA (15–20 ng/μl). The forward and reverse primers were appended at the 5′ end with 18 bp Universal Tag (Unitag) 1 and 2, respectively (Table S1). PCR conditions were 98 °C for 30 s, 25 cycles of denaturation at 98 °C for 10 s, annealing at 56 °C for 20 s and elongation at 72 °C for 20 s, and a final extension at 72 °C for 10 min. The PCR product was examined by gel electrophoresis. The second PCR (100 μl) contained 1× HF buffer, 2 μl dNTP Mix, 2 U of Phusion® Hot Start II High-Fidelity DNA polymerase, 500 nM of a forward and reverse primer (equivalent to the Unitag1 and Unitag2 sequences, respectively) that were each appended with an 8 nt sample specific barcode (Ramiro-Garcia et al. 2016) at the 5′ end, and 5 μl PCR product of the first reaction. PCR conditions were similar to those used for the first PCR except for an annealing temperature of 52 °C and a reduced number of 5 cycles. The PCR product was examined by gel electrophoresis, and amplicons were purified with HighPrep™ (Magbio Genomics, USA) and quantified using a Qubit in combination with the dsDNA BR Assay Kit (Invitrogen, USA). Purified PCR products were pooled, underwent adaptor ligation, and were sequenced on a MiSeq platform (GATC-Biotech, Germany). Sequence analysis was performed with NG-Tax, an in-house pipeline (Ramiro-Garcia et al. 2016) in which operational taxonomic units (OTUs) were assigned to taxonomy using uclust (Edgar 2010) in an open reference approach against the SILVA 16S rRNA gene reference database (Quast et al. 2013). Finally, a biom file was generated and sequence data were further analyzed using Quantitative Insights Into Microbial Ecology (QIIME) v1.2 (Caporaso et al. 2010).

### Quantitative PCR

The abundance of the 16S rRNA genes of *Dehalococcoides, Dehalobacter*, *Desulfitobacterium*, *Geobacter,* and total bacteria was determined by qPCR performed on a CFX384 Real-Time system with C1000 Thermal Cycler (Bio-Rad Laboratories, USA) with iQ™ SYBR Green Supermix (Bio-Rad Laboratories, USA). Each DNA sample was analyzed in triplicates. The primers and qPCR amplification programs used in this study were described before for *Dehalobacter* (Atashgahi et al. 2013) and other targets (Sutton et al. 2015). The relative abundance of *Dehalococcoides* in qPCR data was calculated by dividing the number of *Dehalococcoides* 16S rRNA gene copies by the copy number of bacterial 16S rRNA gene copies, multiplied by 100.

### Nucleotide sequences

16S rRNA gene sequences were deposited in the European Nucleotide Archive (ENA) with accession number ERS1082969-ERS1083005 under study PRJEB13024.

## Results

### TeCB dechlorination in sediment microcosms

During 93 days of anoxic cultivation (Fig. 1, step 2), complete depletion of two spikes of 50 μM of the added TeCBs, i.e., either 1,2,3,4-, 1,2,3,5-, or 1,2,4,5-TeCB, was observed in all sediment microcosms with TCBs and DCBs as products (data not shown). In contrast, no decrease of TeCBs was observed and no dechlorination products were detected in the abiotic controls (data not shown).

**Fig. 2:**
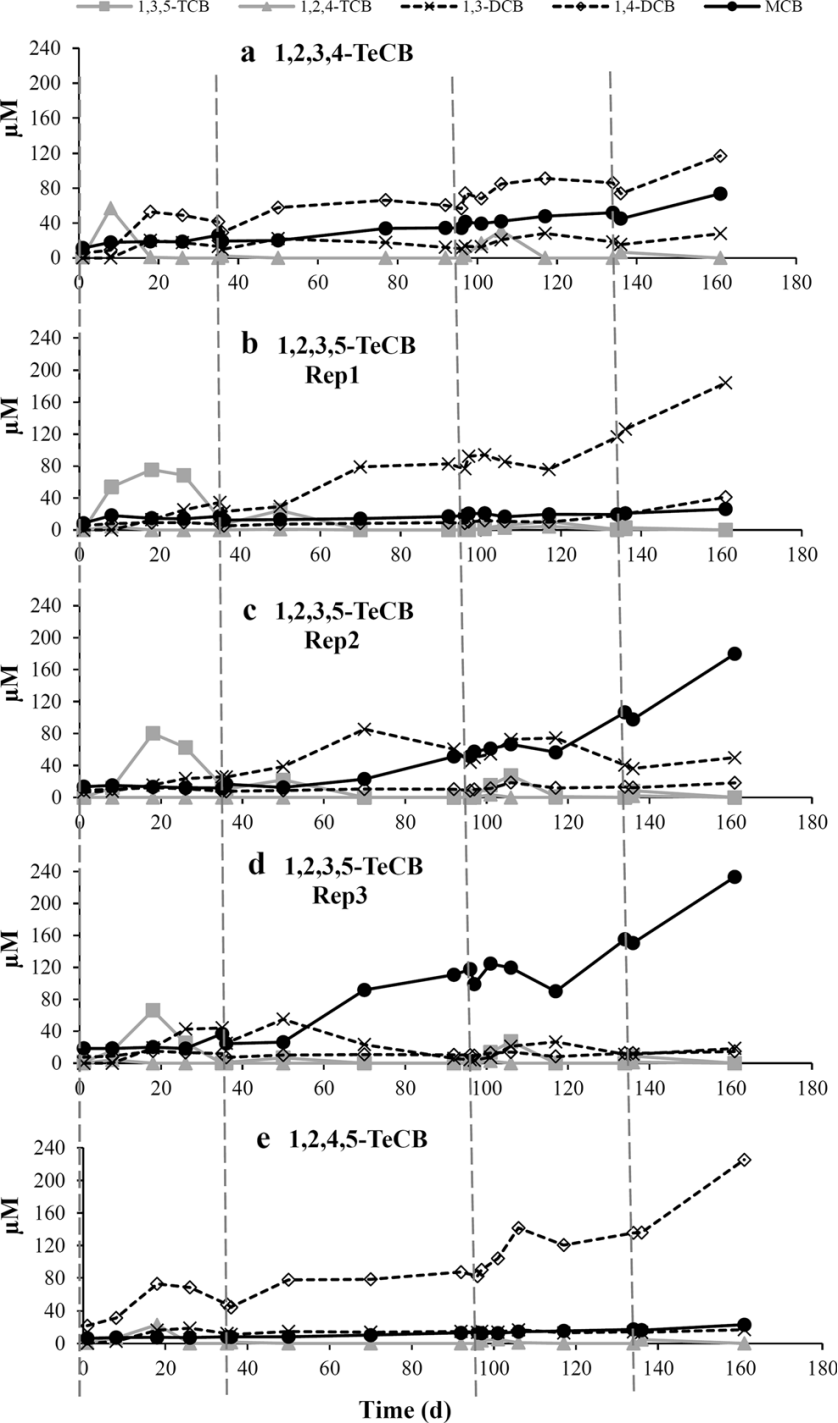
Dechlorination of 1,2,3,4-TeCB (**a**) 1,2,3,5-TeCB (**b–d**, triplicate bottles), and 1,2,4,5-TeCB (**e**) in original microcosms derived from harbor sludge. 50 μM spikes of the respective TeCBs are indicated by *vertical dashed lines*. Concentrations of TeCBs are not shown for the clarity of the figure. Mass balance between parent and daughter products during dechlorination is shown in Table S2

After these microcosms had been stored for 5 years at 4 °C, new spikes of TeCBs were added to recover dechlorination activity. After a short lag phase of 1–8 days, dechlorination of TeCBs was observed in all microcosms (Fig. 2) indicating that long-term storage at cold temperature did not negatively affect the dechlorination activity of the microcosms. 1,2,4-TCB was observed as a transient intermediate during the dechlorination of all three TeCB isomers, whereas 1,3,5-TCB was only detected as an intermediate product of 1,2,3,5-TeCB-dechlorination. All microcosms showed MCB accumulation except for the 1,2,4,5-TeCB-fed microcosm in which 1,4-DCB was detected as the main end product (Fig. 2e). 1,2,3-TCB, 1,2-DCB, and benzene were not detected in any of the microcosms during 161 days of incubation.

### 1,2,4,5-TeCB dechlorination in sediment-free cultures

To achieve sediment-free TeCB-enriched cultures, a 5% inoculum was directly transferred from original TeCB-fed microcosms (Fig. 1, step 4). Dechlorination was observed in the transferred 1,2,4,5-TeCB-fed enrichments (culture A in Fig. 1, step 5) with 1,3- and 1,4-DCB as end products after 66 days of anoxic cultivation, but not in the 1,2,3,4- and 1,2,3,5-TeCB-fed cultures (data not shown).

**Fig. 3:**
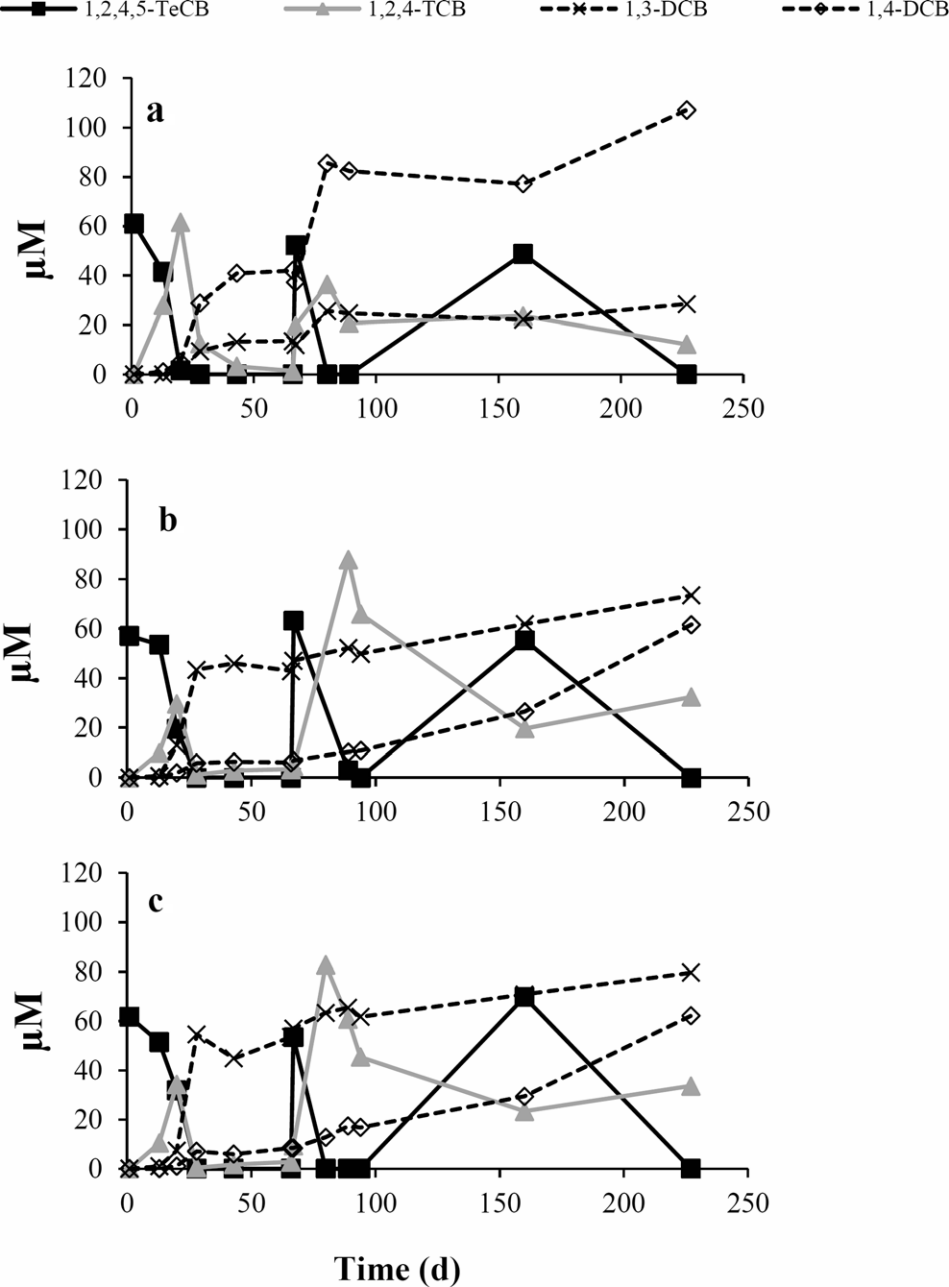
Dechlorination of 1,2,4,5-TeCB in second transfers (Fig. 1, step 6) from the 1,2,4,5-TeCB dechlorinating enrichment derived from harbor sludge (**a**) and 1,2,4,5-TeCB dechlorinating sediment-free enrichments (culture B and C in Fig. 1) derived from river sludge (**b–c**). 50 μM spikes of 1,2,4,5-TeCBs were added at the beginning and on day 66 and 159 of incubation. Data from an individual bottle is shown here as a representative of duplicate cultures, which both followed the same overall dechlorination pattern

In the following, the dechlorination performance of the newly obtained transfer cultures derived from the harbor environment was compared with that of another two sediment-free 1,2,4,5-TeCB-enriching cultures that were previously enriched from river sludge in Kanaal Ieper-Ijzer (Belgium) (Vandermeeren et al. 2014). During incubation of the second transfer, three spikes of 1,2,4,5-TeCB were completely depleted in all transferred enrichments, concomitant with the production of 1,2,4-TCB, 1,4-DCB, and 1,3-DCB (Fig. 3). 1,4-DCB was the dominant end product in harbor derived enrichment (Fig. 3a), which was in line with metabolites observed in the original 1,2,4,5-TeCB-fed microcosm (Fig. 2e). In contrast, slightly more 1,3-DCB was detected in river sludge-derived enrichments (Fig. 3b, c).

### Dechlorination pattern of TeCB isomers and selectivity of the reactions

**Fig. 4:**
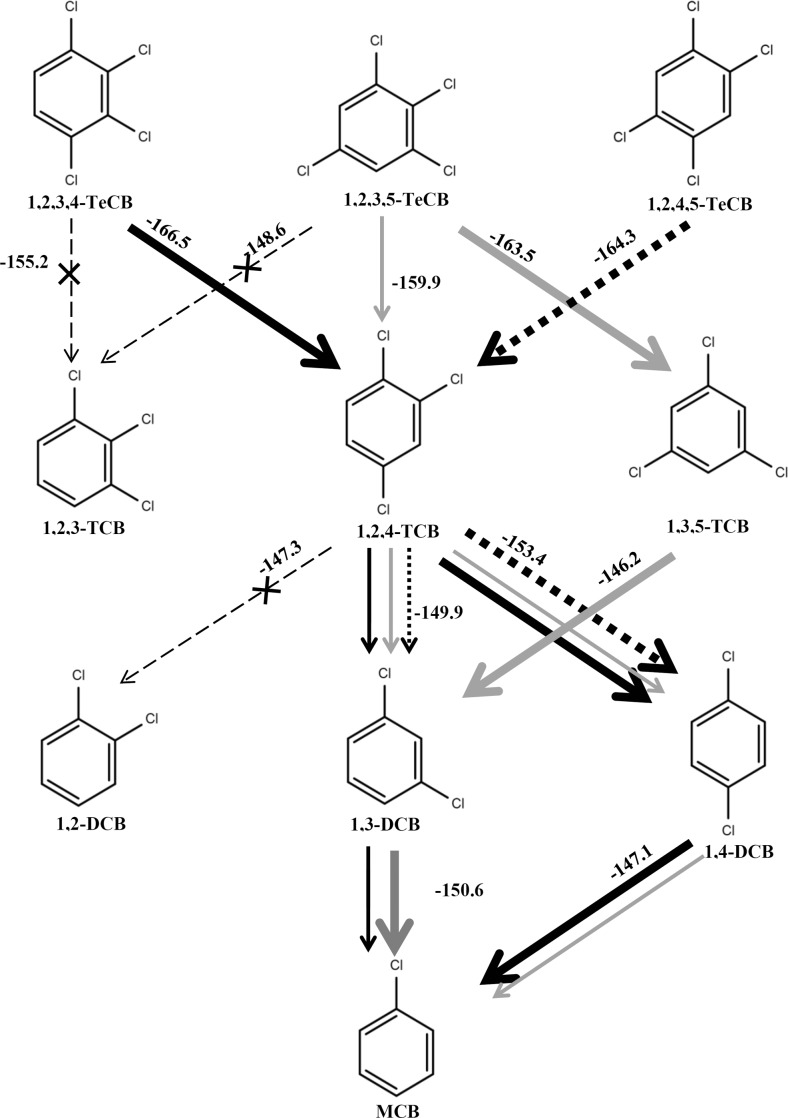
Dechlorination patterns of 1,2,3,4- (*black solid line*), 1,2,3,5- (*gray solid line*) and 1,2,4,5- (*black dotted line*) TeCB in microcosms derived from harbor sludge. *Thin arrows* represent reactions that were detected but were not dominant. Reactions catalyzed preferentially are indicated with *bold arrows*, and reactions not observed in our study are indicated by *dashed lines* with a cross. The ΔG^0^′ (kJ/mol) indicated for each dechlorination reaction is based on previously reported calculations with hydrogen as the electron donor (Dolfing and Keith Harrison 1993)

The reductive removal of doubly, singly, and non-flanked chlorine atoms was noted in the 1,2,3,4- and 1,2,3,5-TeCB-fed microcosms derived from contaminated harbor sludge. In contrast, only singly flanked, but not the non-flanked chlorine, was removed during the dechlorination of 1,2,4,5-TeCB. Based on Gibbs free-energies (ΔG^0^′ under standard condition, kJ/mol) taken from Dolfing and Keith Harrison (1993) calculated with hydrogen as electron donor, the harbor sludge-derived microcosms showed a preference to follow the thermodynamically most favorable dechlorination pathways. For example, 1,2,3,5-TeCB was mainly dechlorinated to 1,3,5-TCB as an intermediate rather than 1,2,4-TCB, and 1,2,3-TCB was not detected, in line with the fact that the latter reaction theoretically yields the least negative free energy (Fig. 4). A similar pattern was observed for the dechlorination of 1,2,3,4-TeCB to 1,2,4-TCB instead of 1,2,3-TCB and further dechlorination of 1,2,4-TCB to 1,4-DCB instead of 1,3-DCB as the main product (Fig. 4).

### Effect of vancomycin addition and vitamin B12 starvation

Vancomycin, an inhibitor of the peptidoglycan synthesis of Gram-positive bacteria, was added to harbor sludge-derived, sediment-free 1,2,4,5-TeCB enrichments to test its effect on TeCB dechlorination. After 21 days of anoxic cultivation, the amended 1,2,4,5-TeCB was completely depleted in control microcosms (Fig. S1a) with 1,4-DCB and 1,3-DCB as the main products. In contrast, in vancomycin treated microcosms, only 36–59% of the 1,2,4,5-TeCB was dechlorinated within the same incubation time, with 1,2,4-TCB as the main product and trace amounts of 1,4-DCB and 1,3-DCB (Fig. S1b). The delayed dechlorination in enrichments supplied with vancomycin suggested (in)direct involvement of vancomycin-sensitive Gram-positive bacteria in 1,2,4,5-TeCB dechlorination. Additionally, vitamin B12 (cofactor of reductive dehalogenase enzymes) starvation was investigated in the presence or absence of vancomycin (Fig. S1c, d). However, no effect was observed on TeCB dechlorination activity, suggesting that the 1,2,4,5-TeCB-enriched microbial communities tested here were capable of de novo vitamin B12 biosynthesis.

### Dynamics of bacterial communities

**Fig. 5:**
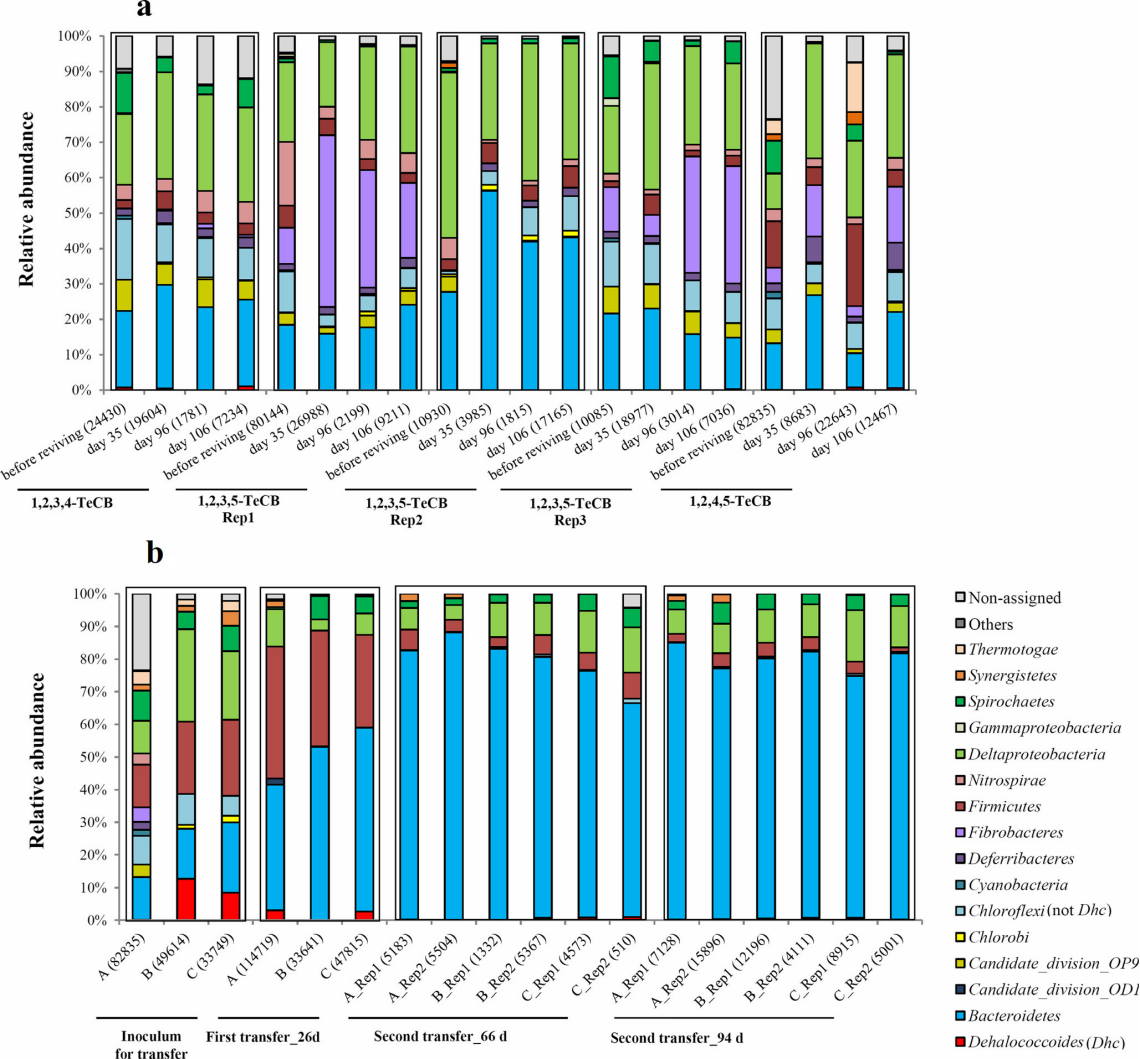
Bacterial 16S rRNA gene-based community dynamics of TeCB-fed microcosms during 161 days of cultivation (**a**) and 1,2,4,5-TeCB dechlorinating enrichments during serial transfers (**b**). **a.** Reps 1, 2, and 3 are triplicate original bottles fed with 1,2,3,5-TeCB. Sampling times are labeled as in Fig. 1 (step 3). **b**. Rep1 and 2 are duplicates. Inoculum and sampling time are labeled as in Fig. 1 (step 6). Data are shown at phylum level, except *Delta*- and *Gammaproteobacteria* that are presented at the class level and the genus *Dehalococcoides* that is shown separately from its corresponding phylum (*Chloroflexi*). The number of reads of each sample is given in *brackets*. Phyla with relative abundance lower than 1% in all samples are summed up as ‘rest’

**Fig. 6:**
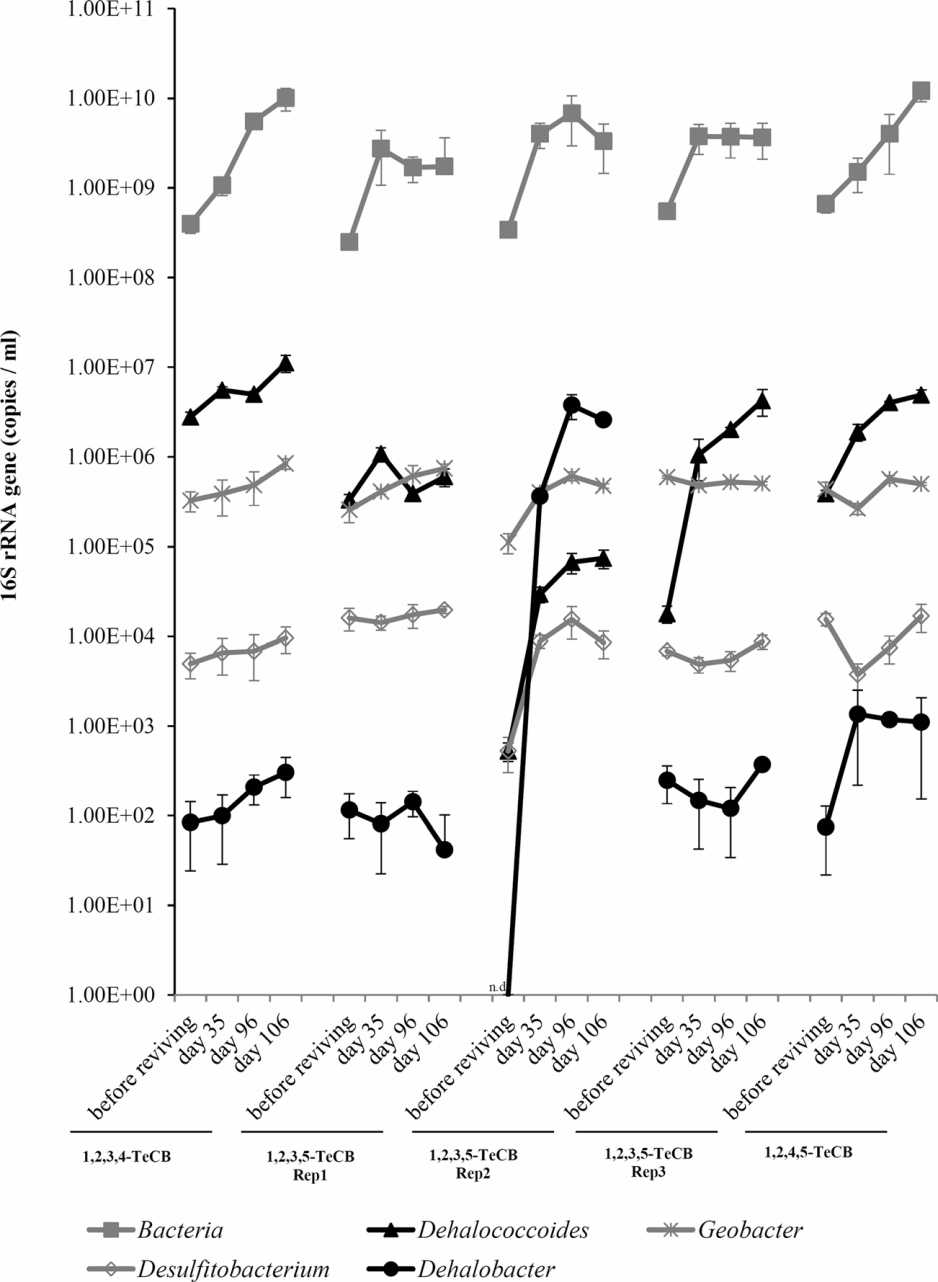
Quantitative PCR (qPCR) targeting 16S rRNA genes of *Dehalobacter*, *Desulfitobacterium*, *Geobacter* and *Dehalococcoides,* and of total bacteria during 161 days of cultivation of TeCB-fed microcosms derived from harbor sludge. Microcosm set up and sampling times are labeled as Fig. 1 (step3). Reps 1, 2, and 3 are triplicate original bottles fed with 1,2,3,5-TeCB. *Error bars* indicate standard deviations of qPCR technical triplicates. *n.d*. nondetectable

During 161 days of cultivation of original TeCB microcosms, *Dehalococcoides* as a potential TeCB dechlorinator was detected at relative abundances below 2% in 1,2,3,4- and 1,2,4,5-TeCB fed microcosms and one (Rep3) of the triplicate 1,2,3,5-TeCB fed microcosms (Fig. 5a). The detected sequence of *Dehalococcoides* (200 bp) showed 100% identity with that of *D. mccartyi* strains CBDB1 and 195 (data not shown). Additionally, diverse bacteria were found in the bacterial community of TeCB-fed microcosms, especially from the phyla *Bacteroidetes* and *Fibrobacteres* and the class *Deltaproteobacteria* (Fig. 5a) that have not reported as OHRB except the deltaproteobacterial *Geobacter lovelyi* that did not show appreciable increase using qPCR analysis (Fig. 6). 16S rRNA genes of *Bacteroidetes* and *Deltaproteobacteria* were detected in all microcosms in the range of 10–56% and 10–47%, respectively. *Fibrobacteres* was mainly enriched in the 1,2,4,5-TeCB-fed microcosm and two of the three replicate 1,2,3,5-TeCB fed microcosms (Rep1, Rep3), whereas it was not detected in the third 1,2,3,5-TeCB fed microcosm during cultivation (Fig. 5a). Differences in bacterial community composition observed among the three replicate 1,2,3,5-TeCB-fed microcosms may have been caused by the heterogeneity of the sediment used for initial inoculation.

During serial transfers of the different 1,2,4,5-TeCB-enriched cultures, members of the *Bacteroidetes* phylum became strongly enriched with an average relative abundance of 79.7% (SD = 3.8, Table S3) at the end of the second transfer (Fig. 5b). *Bacteroidetes* detected in the MiSeq dataset were mainly from the classes *Bacteroidia* (especially from the genus *Petrimonas*) and *Sphingobacteriia* (Table S3). *Petrimonas* was not detected in the TeCB-enriched cultures derived from harbor sludge, in which the *Sphingobacteriia* dominated the bacterial community (Table S3).

### Quantification of potential dechlorinators during TeCB dechlorination

**Fig. 7:**
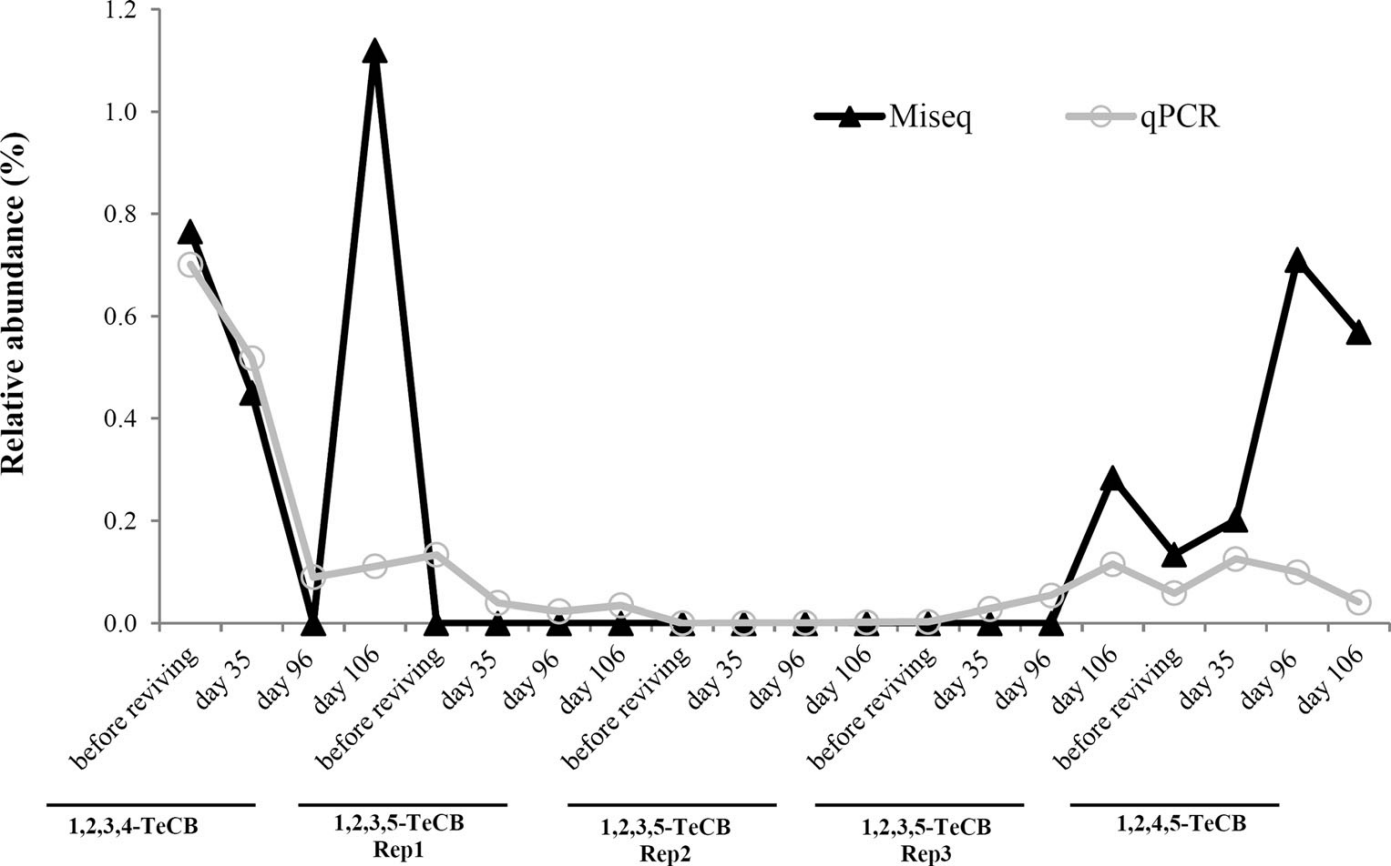
Relative abundance of *Dehalococcoides* in TeCB-fed microcosms during 161 days of cultivation. Reps 1, 2 and 3 are triplicate original bottles fed with 1,2,3,5-TeCB. Sampling times are labeled the same as Fig. 1 (step 3)

Bacterial growth, as measured by bacterial 16S rRNA gene-specific qPCR, was observed in all TeCBs fed microcosms (Fig. 6). Interestingly, in one out of three 1,2,3,5-TeCB fed microcosms (Rep2), the abundance of the genus *Dehalobacter* increased considerably from non-detectable levels before reviving to 2.59E + 06 copies/ml (SD = 3.48E + 05) at day 106 of cultivation, which was higher than the abundance of *Dehalococcoides* 16S rRNA genes with 7.41E + 04 copies/ml (SD = 1.74E + 04). This indicated that in this microcosm, *Dehalobacter* rather than *Dehalococcoides* was the main TeCB dechlorinator. In all other microcosms, a clear dominance of *Dehalococcoides* during cultivation was observed with an average abundance in the range of 3.93E + 05 to 1.12E + 07 copies/ml at the end of the cultivation (Fig. 6). In contrast, 16S rRNA gene copy numbers of *Desulfitobacterium* and *Geobacter* were rather stable (Fig. 6). In all of the 1,2,4,5-TeCB-fed transferred cultures, *Dehalococcoides* was detected with abundances that were more than three orders of magnitude higher than those of *Dehalobacter* (Fig. S2), suggesting a more important role of *Dehalococcoides* in 1,2,4,5-TeCB dechlorination in these enrichments. Overall, the relative abundance of *Dehalococcoides* calculated from qPCR data generally matched with the results derived from the MiSeq dataset, although in several samples higher relative abundance of *Dehalococcoides* was detected via MiSeq (Fig. 7, Fig. S3). Both qPCR and MiSeq results showed decreased relative abundance of *Dehalococcoides* during transfer, which was mainly caused by the increase of average bacterial numbers from 3.05E + 08 copies/ml in the inoculum to 1.98E + 09 copies/ml at 94 days, while the absolute numbers of *Dehalococcoides* remained around 1.0E + 07 copies/ml during serial transfer (Fig. S2).

## Discussion

Dechlorination of 1,2,3,4-, 1,2,3,5-, and 1,2,4,5-TeCB isomers to DCB and/or MCB was observed in anoxic microcosms derived from harbor sludge, and enriched consortia showed selectivity in mediating thermodynamically more favorable steps in the reductive dechlorination of TeCB isomers (Fig. 4). Such preference for thermodynamically more favorable CB dechlorination pathways was shown before in an anaerobic microbial consortium originated from lake sediment (Beurskens et al. 1994), however, in that report, the TeCB isomers were dechlorinated with 1,3,5-TCB or 1,2,4-TCB as the end products. In our study, 1,2,3,5-TeCB was mainly dechlorinated to 1,3,5-TCB with the removal of doubly flanked chlorine, and subsequent removal of the unflanked *meta*-substituted chlorines to produce 1,3-DCB (Fig. 2b) and MCB (Fig. 2c, d). This implies that the enriched consortia from harbor sludge host CB dechlorinator(s) with complementary dechlorination functions. In line with our data, the dechlorination of HCB via 1,2,3,5-TeCB was found to predominate in cultures derived from estuarine sediment with 1,3,5-TCB (Wu et al. 2002) and 1,3-DCB (Pavlostathis and Prytula 2000) as the end products. Furthermore, 1,3,5-TCB production from 1,2,3,5-TeCB was reported before for *D. mccartyi* strains (Adrian et al. 2000; Fennell et al. 2004; Pöritz et al. 2015), however, no further dechlorination of 1,3,5-TCB was observed with those isolates, which might be due to the lack of respective dehalogenases. A different dechlorination pattern of 1,2,3,5-TeCB, with even less favorable thermodynamics, was found in *Dehalobacter* sp. 13DCB1, which predominantly dechlorinated singly flanked *ortho*-substituted chlorine with 1,2,4-TCB as the dominant intermediate leading to the formation of 1,4-DCB as the main end product and small amounts of MCB (Nelson et al. 2014). 1,2,4-TCB and 1,4-DCB were also detected in our 1,2,3,5-TeCB amended microcosms, albeit not as dominant dechlorination products (Fig. 4). Up to date, the dechlorination of 1,2,3,5-TeCB with the removal of unflanked chlorine to produce 1,2,3-TCB has not been reported.

1,2,3-TCB was shown to be the only product of 1,2,3,4-TeCB dechlorination by *Dehalobacter* strain 12DCB1 and 14DCB1 (Nelson et al. 2014) and could be further dechlorinated to 1,3-DCB by *D. mccartyi* strain DCMB5 (Pöritz et al. 2015). However, those metabolites were not detected in our study (Fig. 2a), probably because 1,2,3-TCB production was thermodynamically less favorable compared to the reductive removal of doubly flanked chlorine from 1,2,3,4-TeCB that leads to 1,2,4-TCB formation (Fig. 4).

Furthermore, 1,2,4-TCB was the only TCB detected in the 1,2,4,5-TeCB amended microcosms that was predominantly dechlorinated to 1,4-DCB with slightly more negative change of Gibbs free energy than formation of 1,3-DCB by the microcosm originated from contaminated harbor sludge (Fig. 4). In contrast, more 1,3-DCB than 1,4-DCB was produced via 1,2,4-TCB in the transferred cultures derived from Kanaal Ieper-Ijzer sludge (Fig. 3b, c), indicating the impact of inoculum source on the preference for the prevailing biotransformation pathway. The formation of 1,2-DCB from 1,2,4-TCB with the removal of unflanked meta-chlorine and lowest energy release was neither observed in our microcosms (Fig. 4) nor in other reported enrichment cultures (Middeldorp et al. 1997; Ramanand et al. 1993; Vandermeeren et al. 2014; Zhou et al. 2015) or in studies using CB dechlorinating isolates (Adrian et al. 2000; Fennell et al. 2004; Nelson et al. 2014; Pöritz et al. 2015).

We detected *Dehalococcoides* and *Dehalobacter* in all TeCB-fed microcosms as putative TeCB dechlorinators. In most of the microcosms, the growth of *Dehalococcoides* during 161 days of cultivation was associated with the addition of new spikes of the different TeCB substrates and subsequent TeCB dechlorination (Figs 2 and 6). However, no *Dehalococcoides* isolates have been previously reported to dechlorinate 1,2,3,5-TeCB to 1,4-DCB via 1,2,4-TCB or to dechlorinate any DCBs to MCB. Hence, the detected 1,2,4-TCB and 1,4-DCB in one of the 1,2,3,5-TeCB-fed microcosm among triplicates and the MCB increase in all the 1,2,3,4- and 1,2,3,5-TeCB-fed microcosms were possibly due to the dechlorination activity of *Dehalobacter* or other unknown dechlorinators. Except for one replicate microcosm, the obtained growth yields of *Dehalococcoides* by reductive dechlorination of amended TeCB isomers in original microcosms derived from harbor sludge (1.7 × 10^10^ to 2.4 × 10^10^ cells per mmol chloride released, Table S4) were comparable to the values reported for reductive dehalogenation of 2,3-dichlorophenol (7.6 × 10^10^ cells per mmol chloride released) (Adrian et al. 2007) and tetra- and trichloroethene (3.9 × 10^9^ cells per mmol chloride released) (Marco-Urrea et al. 2011) by a pure culture of *Dehalococcoides mccartyi* strain CBDB1. This indicates that *Dehalococcoides* was mainly responsible for the reductive dechlorination patterns observed in these microcosms. However, *Dehalococcoides* growth yield was lower in one of the 1,2,3,5-TeCB dechlorinating microcosms (7.9 × 10^8^ cells per mmol chloride released). Such low growth yields were previously reported from biostimulated aquifer microcosms dechlorinating trichloroethene (Schneidewind et al. 2014). Hence, we cannot exclude reductive dechlorination by other OHRB not quantified in this study or even co-metabolic reductive dechlorination (Assafanid et al. 1992, Gantzer and Wackett 1991).

*Dehalococcoides* and *Dehalobacter* both use hydrogen as the sole electron donor for reductive dehalogenation and are corrinoid auxotrophs (Holliger et al. 1998; Löffler et al. 2013; Nelson et al. 2014; Rupakula et al. 2015). Hence, the lack of a negative effect of vitamin B12 starvation on the 1,2,4,5-TeCB dechlorination capability of the transferred cultures indicated the presence of corrinoid producers in the microbial community that fulfill nutritional requirements of the TeCB dechlorinators. Considerable inhibition of dechlorination activity by vancomycin was observed in our study, and the 1,2,4,5-TeCB dechlorination pathway was changed in transferred cultures (Fig. S1). This sensitivity to vancomycin was observed before in HCB and 1,3,5-TCB fed microcosms derived from anoxic sediments (Duan and Adrian 2013), and therefore indicated the (in)direct involvement of vancomycin-sensitive bacteria in 1,2,4,5-TeCB dechlorination in transferred cultures. *Dehalococcoides* members lack a typical bacterial cell wall and therefore are not sensitive to vancomycin (Löffler et al. 2013), and members of *Dehalobacter* were found resistant to vancomycin before (Nelson et al. 2011) which might explain why vancomycin treatment did not fully abolish dechlorination activity.

Bacteria, belonging to the genera *Desulfitobacterium* and *Geobacter,* were also detected in the TeCB-fed microcosms by qPCR (Fig. 6). Although high abundance of *Geobacter* was previously found in HCB dechlorinating consortia (Zhou et al. 2015), and *Desulfitobacterium*-like populations predominated CBs-dechlorinating bacterial communities (Vandermeeren et al. 2014), these two genera have not been previously reported to dechlorinate CB, and hence no conclusion can be drawn on their (in)direct role in dechlorination of TeCB isomers and/or corresponding daughter products. Moreover, *Desulfitobacterium* and *Geobacter* are known as versatile facultative OHRB (Lovley et al. 2011; Villemur et al. 2006) that can use lactate as electron donor and other electron acceptors like sulfate that was also included in the media used in our study.

In our study, members of the phylum *Fibrobacteres* were detected in all TeCB-fed microcosms except one of three 1,2,3,5-TeCB-fed microcosms, with relative abundances ranging up to 49.5% (Fig. 5). Members of this phylum are known as plant polymer-degrading bacteria and were reported before from anoxic cellulose-fed digesters and herbivore guts (Rahman et al. 2015). Even though no evidence has been shown for their (in)direct involvement in reductive dechlorination of organohalides, they were detected before in contaminated sites, including a hexahydro-1,3,5-trinitro-1.3.5-triazine (RDX) contaminted vadose zone (Cupples 2013; Ronen et al. 2008) and soils contaminated with cadmium (Jeong et al. 2013) and crude oil (Abbasian et al. 2016). High relative abundance of sulfate-reducing *Deltaproteobacteria*, mainly from the *Desulfobacteraceae* family, with relative abundances ranging between 5 and 25.9% was detected in all TeCB-fed microcosms during 161 days of cultivation (Fig. 5a). Sulfate reducing members of the *Deltaproteobacteria* have previously been suggested to support *Dehalococcoides* by providing essential corrinoids at batch (Hug et al. 2012) and field (Atashgahi et al. 2016) scales and therefore may have a stimulatory effect on dechlorination (Sutton et al. 2015).

Increase of *Bacteroidetes* abundance has previously been reported after in situ bioaugmentation to treat trichloroethene (TCE)-contaminated groundwater, which indicated a potential syntrophy and/or synergistic relationship between *Bacteroidetes* and chlorinated ethene dechlorinators (Pérez-de-Mora et al. 2014). *Bacteroidetes* were also found highly enriched in our study, especially from the genus *Petrimonas* and the class *Sphingobacteriia*. Members of the genus *Petrimonas* are known as anaerobic fermentative bacteria and were previously isolated from a biodegraded oil reservoir and confirmed as lactate fermentator (Grabowski et al. 2005). Furthermore, *Petrimonas* was found predominant in a laboratory upflow anoxic sludge blanket (UASB) reactor treating TCE containing wastewater supplied with glucose and lactate as substates, and *Petrimonas* showed a positive correlation with TCE removal efficiency (Zhang et al. 2015). In our study, *Petrimonas* was mainly detected in the transferred cultures derived from river sludge collected in Kanaal Ieper-Ijzer (Table S3), but not from transfers derived from the harbor sludge, in which *Sphingobacteriia* dominated the bacterial community (Table S3). *Sphingobacteriia* were reported before to use lactate as carbon source (Shivaji et al. 1992), which might explain their considerable growth found in our study. Bacterial clones affiliated with *Sphingobacteriia* were detected before in a TCE-degrading community after in situ biostimulation with lactate (Macbeth et al. 2004), and sequences of *Sphingobacteriia* (in family WCHB1–69) were detected in a dioxin-dehalogenating enrichment derived from contaminated freshwater sediment with acetate as carbon source (Bunge et al. 2008) and in RDX-contaminated groundwater after biostimulation with acetate (Livermore et al. 2013). In our study, a strong increase of the *Bacteroidetes* population in the transferred cultures suggested a potential link (e.g., syntrophy and/or synergy) between lactate-utilizing *Bacteroidetes* and CB dechlorinators. Additionally, *Firmicutes*, all from the *Clostridiales* order, were detected in the transferred cultures, and especially in the first transfer (Fig. 5b). In addition to known organohalide-respiring *Clostridiales* belonging to the genera *Dehalobacter* and *Desulfitobacterium,* other *Clostridiales* were reported at high relative abundances in microcosms enriched with CBs (Vandermeeren et al. 2014), chlorinated ethane (Merlino et al. 2015), chlorinated ethene (Cichocka et al. 2010), and chlorinated phenols (Zhang et al. 2012) as electron acceptor and lactate as the electron donor, which suggested their role as important co-existing microbial guilds in organohalide-contaminated environments and a potential partnership between corrinoid-producing *Clostridiales* that are most probably non-dechlorinating and CBs dechlorinators (Men et al. 2013). One of the genera within the *Clostridiales* order found in this study, *Sedimentibacter*, was previously reported in co-culture with *Dehalobacter* sp. E1 and was postulated to support β-hexachlorocyclohexane (HCH) dechlorination by providing essential co-factors (e.g., corrinoid) (Maphosa et al. 2012; van Doesburg et al. 2005). *Sedimentibacter* alone was not able to survive in HCH-fed media (van Doesburg et al. 2005), and therefore, OHRB as detoxificator of chlorinated contaminants may have stimulatory impact on the growth of other anaerobic community members.

In summary, in this study we report the reductive dechlorination of three TeCB isomers in anoxic microcosms enriched from contaminated harbor sludge. Thermodynamically favorable dechlorinating pathways were found to be selectively followed by the TeCB-fed microcosms. Additionally, growth of *Dehalobacter* and *Dehalococcoides* was found to be associated with CB dechlorination based on 16S rRNA gene-targeted qPCR supporting their role in the observed reductive dehalogenation. Bacterial community profiling showed diverse putative non-dechlorinating bacteria, some of which may have stimulated TeCB dechlorination by providing essential nutrients, including electron donor and cofactors. Overall, these results provide further insight into the potential CB dechlorination in contaminated harbor environments and the interaction between TeCB dechlorinators and co-existing non-dechlorinators.

## Electronic supplementary material

ESM 1 (PDF 201 kb)
