# Supplementary material for: Dechlorination of three tetrachlorobenzene isomers by contaminated harbor sludge-derived enrichment cultures follows thermodynamically favorable reactions
Source: Appl Microbiol Biotechnol. 2016 Dec 1;101(6):2589–601. doi: 10.1007/s00253-016-8004-8 (PMC5320011; doi:10.1007/s00253-016-8004-8)
Supplement: Supplementary file 1 — (PDF 201 kb) [file 253_2016_8004_MOESM1_ESM.pdf]

Title: Dechlorination of three tetrachlorobenzene isomers by contaminated harbor sludge-derived enrichment cultures follows thermodynamically favorable reactions

Journal name: Applied Microbiology and Biotechnology

Author name: Yue Lu[1], Javier Ramiro-Garcia[12], Pieter Vandermeeren[3], Steffi Herrmann[3], Danuta Cichocka[3], Dirk Springael[3] Siavash Atashgahi[1] and Hauke Smidt[1*]

[1] Laboratory of Microbiology, Wageningen University, Wageningen, The Netherlands

[2] Laboratory of Systems and Synthetic Biology, Wageningen University, Wageningen, The Netherlands

[3] Division of Soil and Water Management, KU Leuven, Leuven, Belgium

[*]E-mail: hauke.smidt@wur.nl

Table S1 Overview of the sequences of the MiSeq primers and Unitags

Primer[a]	Oligonucleotide sequence (5´-3´)[b]	Reference
27F-DegS	GTTYGATYMTGGCTCAG	(van den Bogert et al. 2011)
338R-I	GCWGCCTCCCGTAGGAGT	(Daims et al. 1999)
338R-II	GCWGCCACCCGTAGGTGT	(Daims et al. 1999)
Unitag1	GAGCCGTAGCCAGTCTGC	(Tian et al. 2016)
Unitag2	GCCGTGACCGTGACATCG	(Tian et al. 2016)

[a] Primer names may not correspond to original publication

[b] M = A or C; R = A or G; W = A or T; Y = C or T

Table S2 Mass balance between parent and daughter products during reductive dechlorination of 1,2,3,4-TeCB (a), 1,2,3,5-TeCB (b-d, triplicate bottles), and 1,2,4,5-TeCB (e) in original microcosms derived from harbor sludge. The concentrations of CBs detected on Day 1 are residues from previous incubation. Mass balance is calculated subtracting the cumulative concentration of CBs products detected on Day 161 from the concentration of CBs detected on Day 1 plus the supplied TeCB substrates during four spikes. Positive values of mass balance indicate the balance loss during TeCB reductive dechlorination over 161 days

	a. 1,2,3,4-TeCB		b. 1,2,3,5-TeCB Rep1		c. 1,2,3,5-TeCB Rep2		d. 1,2,3,5-TeCB Rep3		e. 1,2,4,5-TeCB	
	Day 1 (μmol)	Day 161 (μmol)	Day 1 (μmol)	Day 161 (μmol)	Day 1 (μmol)	Day 161 (μmol)	Day 1 (μmol)	Day 161 (μmol)	Day 1 (μmol)	Day 161 (μmol)
1,3,5-TCB	0.00	0.00	0.00	0.00	0.00	0.00	0.12	0.00	0.00	0.00
1,2,4-TCB	0.07	0.00	0.00	0.00	0.00	0.00	0.00	0.00	0.05	0.00
1,4-DCB	0.39	5.50	0.37	1.93	0.63	0.85	0.54	0.69	1.53	10.59
1,3-DCB	0.00	1.31	0.00	8.66	0.48	2.34	0.00	0.87	0.00	0.79
MCB	0.79	3.46	0.60	1.23	0.94	8.45	1.30	10.97	0.44	1.08
Cumulative CBs product (μmol)	1.25	10.26	0.97	11.82	2.05	11.65	1.84	12.53	2.01	12.46
Supplied TeCB (μmol)		10.94		10.94		10.94		10.94		10.94
Mass balance (μmol)		1.92		0.09		1.34		0.24		0.49

Table S3 Miseq sequences assigned to the phylum *Bacteroidetes* as observed in bacterial 16S rRNA gene-based community composition data of 1,2,4,5-TeCB-enriched cultures during serial transfers. A is the 1,2,4,5-TeCB-enriched culture derived from harbor sludge; B and C are 1,2,4,5-TeCB-enriched sediment-free cultures derived from river sludge. Inoculum and sampling time are labeled as in Fig 1 (step 6). Rep1 and 2 are duplicates. Taxa with relative abundance lower than 3% in all samples are summed up and shown as ‘rest’. n.d.: nondetectable.

Taxon_*Bacteroidetes*			**Inoculum for serial transfer**			**First transfer_26d**			**Second transfer_66 d**						**Second transfer_94 d**					
Class	Family	Genus	**A**	**B**	**C**	**A**	**B**	**C**	**A _Rep1**	**A _Rep2**	**B _Rep1**	**B _Rep2**	**C _Rep1**	**C _Rep2**	**A _Rep1**	**A _Rep2**	**B _Rep1**	**B _Rep2**	**C _Rep1**	**C _Rep2**
Bacteroidia	*Porphyromonadaceae*	*Petrimonas*	n.d.	2.6	7.3	n.d.	9.8	19.6	n.d.	n.d.	27.7	20.9	29.8	19.8	n.d.	n.d.	20.2	19.3	27.8	33.8
		Proteiniphilum	n.d.	1.2	1.6	n.d.	4.2	2.6	n.d.	n.d.	n.d.	0.4	1.2	1.6	n.d.	n.d.	0.4	0.5	1.0	0.9
Sphingobacteriia	WCHB1-69	-	n.d.	6.8	7.9	38.3	35.4	30.7	80.3	84.1	46.0	52.2	37.5	34.5	82.2	68.7	49.4	53.1	39.3	38.5
vandinHA17	-	-	12.0	2.7	3.6	0.3	0.6	0.8	2.1	4.1	8.9	5.4	5.3	3.1	2.7	8.1	9.0	7.4	5.0	6.8
rest			1.1	1.8	1.1	n.d.	3.1	2.5	n.d.	n.d.	0.5	1.0	1.9	6.5	n.d.	n.d.	0.6	1.3	0.9	1.7
total *Bacteroidetes*			13.1	15.2	21.5	38.5	53.1	56.2	82.3	88.2	83.1	79.9	75.6	65.5	84.8	76.8	79.6	81.5	74.1	81.6

Table S4 Growth yield (cells per mmol chloride released) of *Dehalococcoides* during the dechlorination of 1,2,3,4-TeCB (a), 1,2,3,5-TeCB (b and d), and 1,2,4,5-TeCB (e) in original microcosms derived from harbor sludge over 106 days of incubation. The samples taken before adding TeCBs to the original bottles are indicated as 'before reviving'. One and five 16S rRNA copies per cell were considered for *Dehalococcoides* (Löffler et al. 2013) and *Dehalobacter* (Kruse et al. 2013; Wang et al. 2014) for calculation, respectively. Due to the low abundance of *Dehalobacter* in bottle a-b and c-d, its contribution to CBs dechlorination is assumed negligible.

	Dehalococcoides				*Dehalococcoides* and *Dehalobacter*
	a.1,2,3,4-TeCB	b.1,2,3,5-TeCB Rep1	d.1,2,3,5-TeCB Rep3	e.1,2,4,5-TeCB	c.1,2,3,5-TeCB Rep2
before reviving-day 106	2.4E+10	7.9E+08	1.9E+10	1.7E+10	2.2E+09

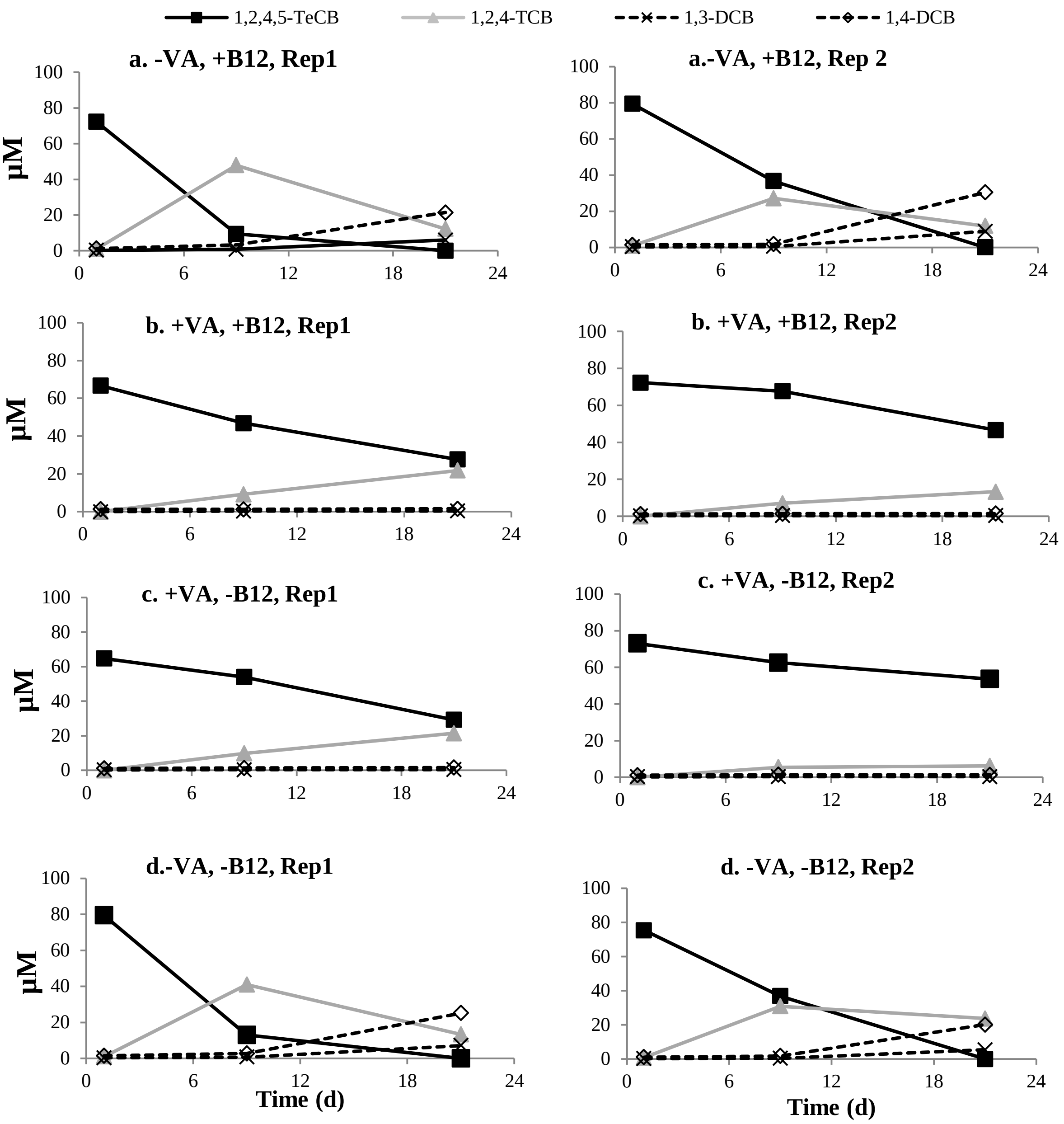

Fig. S1 The effects of vancomycin addition and/or vitamin B12 starvation on the dechlorination activity of sediment-free 1,2,4,5-TeCB-enriched cultures derived from harbor sludge. Rep1 and 2 are replicates. +/- represents addition/deficiency of certain component in the medium

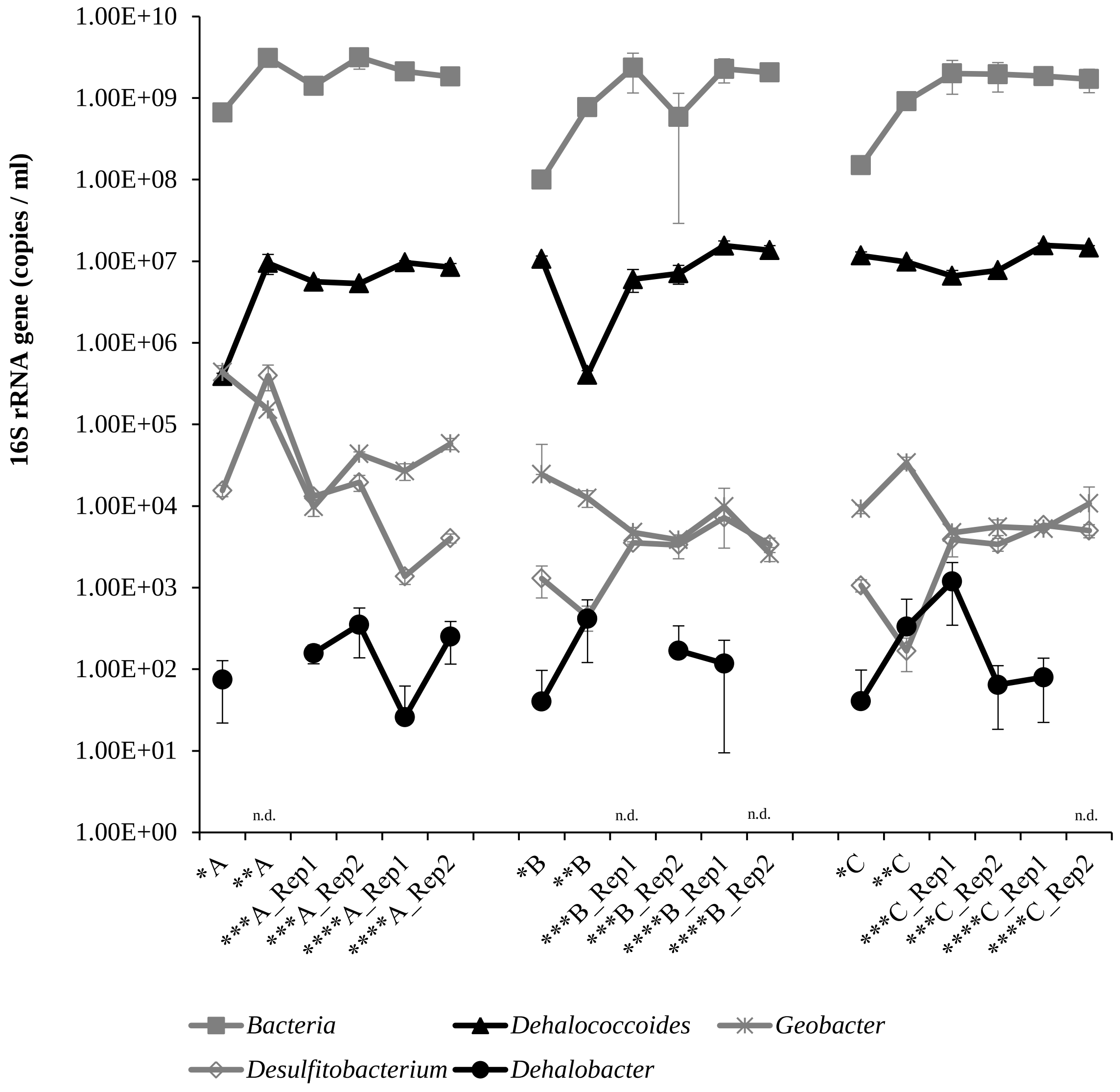

Fig. S2 Quantitative PCR (qPCR) targeting 16S rRNA genes of *Dehalobacter*, *Desulfitobacterium*, *Geobacter*, *Dehalococcoides* and total *Bacteria* during serial transfers of 1,2,4,5-TeCB-enriched cultures. A, is the 1,2,4,5-TeCB-enriched culture derived from harbor sludge; B and C are 1,2,4,5-TeCB-enriched sediment-free cultures derived from river sludge. Symbols for sampling times are: Inoculum for transfer (*), First transfer_26d (**), Second transfer_66d (***) and Second transfer_94d (****). Inoculum and sampling time are indicated as in Fig 1 (step 6). Rep1 and 2 are duplicates. Error bar is the standard deviation of technical triplicates of qPCR. n.d.: nondetectable

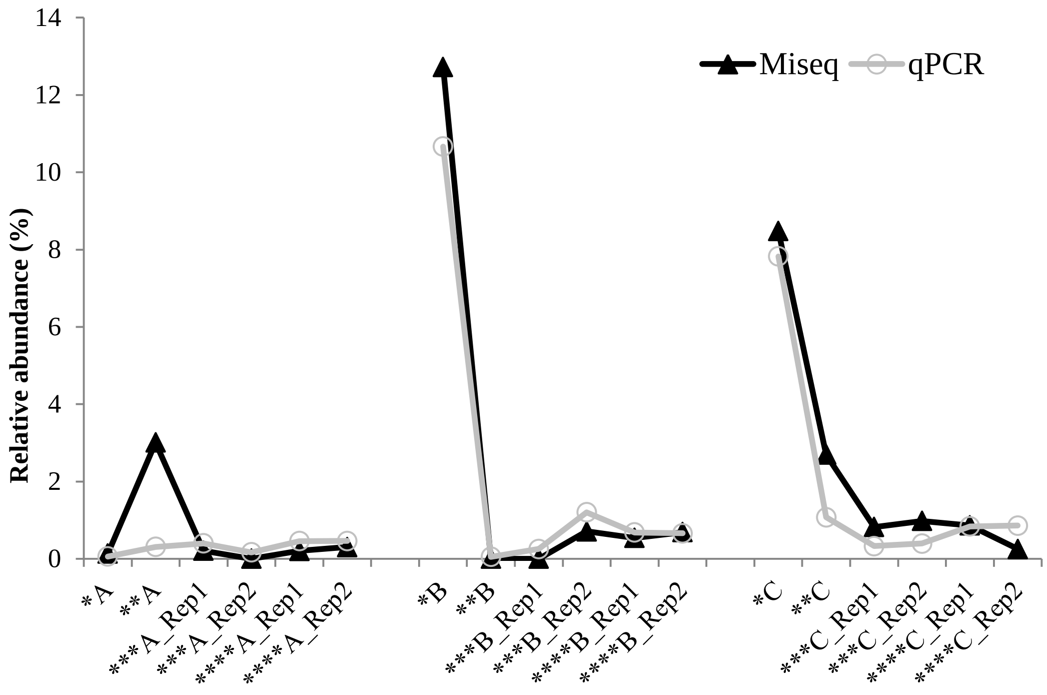

Fig. S3 Relative abundance of *Dehalococcoides* in 1,2,4,5-TeCB dechlorinating enrichments during serial transfers. Symbols for sampling times are: Inoculum for transfer (*), First transfer_26d (**), Second transfer_66d (***) and Second transfer_94d (****). Inoculum and sampling time are indicated as in Fig 1 (step 6). Rep1 and 2 are duplicates.

References

Daims H, Brühl A, Amann R, Schleifer KH, Wagner M (1999) The domain-specific probe EUB338 is insufficient for the detection of all bacteria: development and evaluation of a more comprehensive probe set. Syst Appl Microbiol 22:434-444

Kruse T, Maillard J, Goodwin L, Woyke T, Teshima H, Bruce D, Detter C, Tapia R, Han C, Huntemann M, Wei CL, Han J, Chen A, Kyrpides N, Szeto E, Markowitz V, Ivanova N, Pagani I, Pati A, Pitluck S, Nolan M, Holliger C, Smidt H (2013) Complete genome sequence of *Dehalobacter restrictus* PER-K23^T. Stand Genomic Sci 8:375-388

Löffler FE, Yan J, Ritalahti KM, Adrian L, Edwards EA, Konstantinidis KT, Müller JA, Fullerton H, Zinder SH, Spormann AM (2013) *Dehalococcoides mccartyi* gen. nov., sp. nov., obligately organohalide-respiring anaerobic bacteria relevant to halogen cycling and bioremediation, belong to a novel bacterial class, *Dehalococcoidia* classis nov., order *Dehalococcoidales* ord. nov. and family *Dehalococcoidaceae* fam. nov., within the phylum *Chloroflexi*. Int J Syst Evol Microbiol 63:625-635.

Tian L, Scholte J, Borewicz K, van den Bogert B, Smidt H, Scheurink AJ, Gruppen H, Schols HA (2016) Effects of pectin supplementation on the fermentation patterns of different structural carbohydrates in rats. Mol Nutr Food Res 60:2256-2266

van den Bogert B, de Vos WM, Zoetendal EG, Kleerebezem M (2011) Microarray analysis and barcoded pyrosequencing provide consistent microbial profiles depending on the source of human intestinal samples. Appl Environ Microbiol 77:2071-2080

Wang S, Zhang W, Yang KL, He J (2014) Isolation and characterization of a novel *Dehalobacter* species strain TCP1 that reductively dechlorinates 2,4,6-trichlorophenol. Biodegradation 25:313-323
